# Complete genome sequence of *Rhodospirillum rubrum* type strain (S1^T^)

**DOI:** 10.4056/sigs.1804360

**Published:** 2011-06-30

**Authors:** A. Christine Munk, Alex Copeland, Susan Lucas, Alla Lapidus, Tijana Glavina Del Rio, Kerrie Barry, John C. Detter, Nancy Hammon, Sanjay Israni, Sam Pitluck, Thomas Brettin, David Bruce, Cliff Han, Roxanne Tapia, Paul Gilna, Jeremy Schmutz, Frank Larimer, Miriam Land, Nikos C. Kyrpides, Konstantinos Mavromatis, Paul Richardson, Manfred Rohde, Markus Göker, Hans-Peter Klenk, Yaoping Zhang, Gary P. Roberts, Susan Reslewic, David C. Schwartz

**Affiliations:** 1 Los Alamos National Laboratory, Bioscience Division, Los Alamos, New Mexico, USA; 2 DOE Joint Genome Institute, Walnut Creek, California, USA; 3 University of California San Diego, La Jolla, California, USA; 4 Lawrence Livermore National Laboratory, Livermore, California, USA; 5 HZI – Helmholtz Centre for Infection Research, Braunschweig, Germany; 6 DSMZ – German Collection of Microorganisms and Cell Cultures, Braunschweig, Germany; 7 University of Wisconsin-Madison, Madison, Wisconsin, USA

**Keywords:** facultatively anaerobic, photolithotrophic, mesophile, Gram-negative, motile, *Rhodospirillaceae*, *Alphaproteobacteria*, DOEM 2002

## Abstract

*Rhodospirillum rubrum* (Esmarch 1887) Molisch 1907 is the type species of the genus *Rhodospirillum*, which is the type genus of the family *Rhodospirillaceae* in the class *Alphaproteobacteria*. The species is of special interest because it is an anoxygenic phototroph that produces extracellular elemental sulfur (instead of oxygen) while harvesting light. It contains one of the most simple photosynthetic systems currently known, lacking light harvesting complex 2. Strain S1^T^ can grow on carbon monoxide as sole energy source. With currently over 1,750 PubMed entries, *R. rubrum* is one of the most intensively studied microbial species, in particular for physiological and genetic studies. Next to *R. centenum* strain SW, the genome sequence of strain S1^T^ is only the second genome of a member of the genus *Rhodospirillum* to be published, but the first type strain genome from the genus. The 4,352,825 bp long chromosome and 53,732 bp plasmid with a total of 3,850 protein-coding and 83 RNA genes were sequenced as part of the DOE Joint Genome Institute Program DOEM 2002.

## Introduction

Strain S1^T^ (= ATCC 11170 = DSM 467) is the neotype strain of the species *Rhodospirillum rubrum*, which is the type species of the genus *Rhodospirillum*. The genus name is derived from the ancient Greek term *rhodon*, meaning *rose*, and the Latin *spira*, meaning *coil*. *Rubrum* is Latin for red. Currently *R. rubrum* is one out of only four species with a validly described name in this genus. Strain S1^T^ (van Niel) was designated as the neotype strain for *R. rubrum* by Pfennig and Trüper in 1971 [1], with the description of the strain in complete agreement with the species description given by van Niel in 1944 [2] for the initial deposition at the American Type Culture Collection (ATCC). A comparative genomic analysis with the only other publicly available rhodospirillal genome was recently published by Lu *et al.* [3]. Here we present a summary classification and a set of features for *R. rubrum* S1^T^, together with the description of the complete genomic sequencing and annotation.

## Classification and features

**Figure 1:**
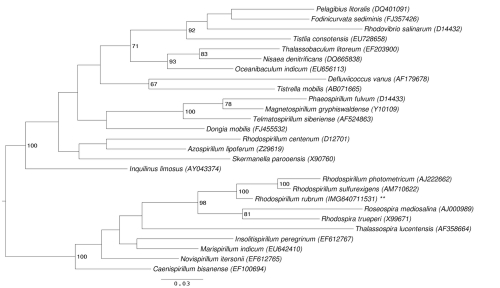
Phylogenetic tree highlighting the position of *R. rubrum* S1^T^ relative to the other type strains within the family *Rhodospirillaceae*. The 16S rRNA accessions were selected from the most recent release of the All-Species-Living-Tree-Project [4] as far as possible. The tree was inferred from 1,361 aligned characters [5,6] of the 16S rRNA gene sequence under the maximum likelihood criterion [7]. Rooting was done initially using the midpoint method [8] and then checked for its agreement with the current classification (Table 1). The branches are scaled in terms of the expected number of substitutions per site. Numbers to the right of bifurcations are support values from 550 bootstrap replicates [9] if larger than 60%. Lineages with type strain genome sequencing projects registered in GOLD [10] are labeled with one asterisk, those also listed as 'Complete and Published' with two asterisks.

Figure 1 shows the phylogenetic neighborhood of *R. rubrum* S1^T^ in a 16S rRNA based tree. The sequences of the four 16S rRNA gene copies in the genome do not differ from each other, and do not differ from the previously published 16S rRNA sequence (X87278), which contains two ambiguous base calls.

**Table 1:** Classification and general features of *R. rubrum* according to the MIGS recommendations [11].

**MIGS ID**	**Property**	**Term**	**Evidence code**
	Current classification	Domain *Bacteria*	TAS [12]
		Phylum ‘*Proteobacteria*’	TAS [13]
		Class *Alphaproteobacteria*	TAS [14,15]
		Order *Rhodospirillales*	TAS [16,17]
		Family *Rhodospirillaceae*	TAS [16,17]
		Genus *Rhodospirillum*	TAS [17-21]
		Species *Rhodospirillum rubrum*	TAS [17,18,22]
		Type strain S1	TAS [1,2]
	Gram stain	negative	NAS
	Cell shape	spiral-shaped	TAS [1]
	Motility	motile	TAS [1]
	Sporulation	not reported	
	Temperature range	mesophile	NAS
	Optimum temperature	25-30°C	NAS
	Salinity	not reported	
MIGS-22	Oxygen requirement	facultative anaerobe	TAS [2]
	Carbon source	numerous 1- and multi-C compounds	TAS [2]
	Energy metabolism	photolithotroph, photoautotroph, aerobic heterotroph, fermentation carbon monoxide	TAS [2,23]
MIGS-6	Habitat	fresh water	NAS
MIGS-15	Biotic relationship	free living	NAS
MIGS-14	Pathogenicity	none	NAS
	Biosafety level	1	TAS [24]
	Isolation	not reported	
MIGS-4	Geographic location	not reported	
MIGS-5	Sample collection time	1941	TAS [2]
MIGS-4.1 MIGS-4.2	Latitude Longitude	not reported	
MIGS-4.3	Depth	not reported	
MIGS-4.4	Altitude	not reported	

Evidence codes - IDA: Inferred from Direct Assay (first time in publication); TAS: Traceable Author Statement (i.e., a direct report exists in the literature); NAS: Non-traceable Author Statement (i.e., not directly observed for the living, isolated sample, but based on a generally accepted property for the species, or anecdotal evidence). These evidence codes are from of the Gene Ontology project [25]. If the evidence code is IDA, the property was directly observed by one of the authors or an expert mentioned in the acknowledgements.

A representative genomic 16S rRNA sequence of strain S1^T^ was compared using NCBI BLAST under default settings (e.g., considering only the high-scoring segment pairs (HSPs) from the best 250 hits) with the most recent release of the Greengenes database [26] and the relative frequencies, weighted by BLAST scores, of taxa and keywords (reduced to their stem [27]) were determined. The five most frequent genera were *Rhizobium* (41.6%), *Rhodospirillum* (30.8%), *Aquaspirillum* (6.2%), *Rhodocista* (4.2%) and *Novosphingobium* (3.5%) (130 hits in total). Regarding the 16 hits to sequences from members of the species, the average identity within HSPs was 98.5%, whereas the average coverage by HSPs was 97.8%. Regarding the five hits to sequences from other members of the genus, the average identity within HSPs was 95.3%, whereas the average coverage by HSPs was 95.0%. Among all other species, the one yielding the highest score was *Rhodospirillum photometricum*, which corresponded to an identity of 96.0% and an HSP coverage of 96.9%. (Note that the Greengenes database uses the INSDC (= EMBL/NCBI/DDBJ) annotation, which is not an authoritative source for nomenclature or classification.) The highest-scoring environmental sequence was AM691104 ('*Rhodobacteraceae* clone EG16'), which showed an identity of 91.7% and an HSP coverage of 97.2%. The five most frequent keywords within the labels of environmental samples which yielded hits were 'ocean' (2.5%), 'microbi' (2.4%), 'soil' (2.1%), 'skin' (1.8%) and 'aquat/rank' (1.8%) (120 hits in total). Environmental samples which yielded hits of a higher score than the highest scoring species were not found.

**Figure 2:**
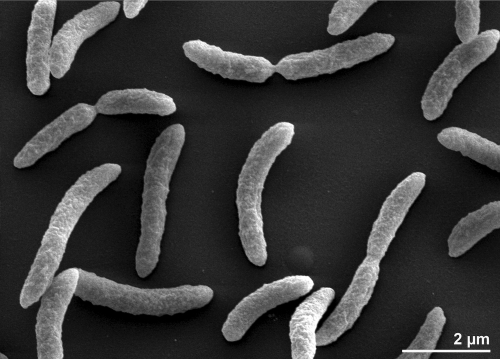
Scanning Electron micrograph of *R. rubrum* S1^T^ generated from a culture of DSM 467

Cells of *R. rubrum* stain Gram-negative, are motile, vibrioid to short spiral-shaped with a size of 0.8-1 µm (Figure 2). Colonies are purple-colored because the cells contain a carotenoid pigment required to gather light energy for photosynthesis. *R. rubrum* does not produce oxygen, but elemental sulfur as a by-product of photosynthesis, using bacteriochlorophyll, which enables the absorbtion of light at wavelengths longer than those absorbed by plants. Strain S1^T^ is a facultative anaerobe that uses alcoholic fermentation under low oxygen conditions, but respiration under aerobic conditions. Photosynthesis is genetically suppressed under aerobic conditions; *R. rubrum* is colorless under these conditions. The regulation of the photosynthetic machinery is still poorly understood, though the organism is phototactic [28]. The RuBisCO (Ribulose-1,5-bisphosphate carboxylase oxygenase) of *R. rubrum* is highly unusual in its simplicity as a homodimer [29].

*R. rubrum* is a well-established model organism for studies on nitrogen fixation and the organism possesses two related but distinct nitrogenase systems that utilize distinct metals at the active site [30]. The post-translational regulation of nitrogenase in *R. rubrum* is relatively unusual in that it utilizes a reversible ADP-ribosylation process [31-35]. The organism has also been used to study bacterial growth on carbon monoxide as an energy source [23], and its carbon monoxide sensor, termed CooA, has been the paradigm for such sensors [36]. *R. rubrum* provides several potential biotechnological applications, e.g. the accumulation of PHB precursors for plastic production in the cell, as well as the production of hydrogen fuel.

### Chemotaxonomy

The composition of the *R. rubrum* cell wall has previously been reported in various publications. The main fatty acids of strain S1^T^ are unbranched, with unsaturated acids C_16:1 w7c_ (34.1%), C_18:1 w7c/12t/9t_ (32.8%) and C_18:1 2OH_ (6.9%) dominating over a minority of saturated acids: C_16:0_ (11.6%) and C_14:0_ (4.0%) [analyzed with a culture of CCUG 17859, www.ccug.se].

## Genome sequencing and annotation

### Genome project history

This organism was selected for sequencing on the basis of the DOE Joint Genome Institute Program DOEM 2002. The genome project is deposited in the Genomes On Line Database [10] and the complete genome sequence is deposited in GenBank. Sequencing, finishing and annotation were performed by the DOE Joint Genome Institute (JGI). A summary of the project information is shown in Table 2.

**Table 2:** Genome sequencing project information

**MIGS ID**	**Property**	**Term**
MIGS-31	Finishing quality	Finished
MIGS-28	Libraries used	Two genomic Sanger libraries: 3 kb pUC18c library, fosmid (40 kb) library
MIGS-29	Sequencing platforms	ABI3730
MIGS-31.2	Sequencing coverage	11.0 × Sanger
MIGS-30	Assemblers	phrap
MIGS-32	Gene calling method	Critica complemented with the output of Glimmer
	INSDC ID	CP000230 (chromosome) CP000231 (plasmid)
	GenBank Date of Release	December 13, 2005
	GOLD ID	Gc00396
	NCBI project ID	58
	Database: IMG	637000241
MIGS-13	Source material identifier	ATCC 11170
	Project relevance	Bioenergy

### Strain history

The history of strain S1^T^ starts with C. B. van Niel (strain ATH 1.1.1, probably 1941) → S.R. Elsden strain S1 → NCI(M)B 8255 → ATCC 11170, from which later on DSM 467, LMG 4362 and CCRC 16403 were derived.

### Growth conditions and DNA isolation

The culture of strain S1^T^, ATCC 11170, used to prepare genomic DNA (gDNA) for sequencing was only 3 transfers away from the original deposit. The culture used to prepare genomic DNA (gDNA) for sequencing, was purified from the original deposit on rich SMN [37] plates, and then grown in SMN liquid medium aerobically. MasterPure Genomic DNA Purification Kit from Epicentre (Madison, WI) was used for total DNA isolation from *R. rubrum*, with a few minor modifications as described previously [38]. One-half to 1 ml of cells was used for DNA isolation. After isopropanol precipitation, DNA was resuspended in 500 µl of 0.1 M sodium acetate and 0.05 M MOPS (pH 8.0), then reprecipitated with 2 volume of ethanol. This step was repeated twice and significantly improved the quality of DNA. The purity, quality and size of the bulk gDNA preparation were assessed by JGI according to DOE-JGI guidelines.

### Genome sequencing and assembly

The genome was sequenced using the Sanger sequencing platform (3 kb and 40 kb DNA libraries). All general aspects of library construction and sequencing performed at the JGI can be found at [39]. The Phred/Phrap/Consed [40] software package was used for sequence assembly and quality assessment. After the shotgun stage, reads were assembled with parallel phrap (High Performance Software, LLC). Possible mis-assemblies were corrected with Dupfinisher or transposon bombing of bridging clones (Epicentre Biotechnologies, Madison, WI) [41]. Gaps between contigs were closed by editing in Consed, custom primer walk or PCR amplification. A total of 847 additional custom primer reactions were necessary to close gaps and to raise the quality of the finished sequence. The completed genome sequence contains 62,976 reads, achieving an average of 11-fold sequence coverage with an error rate of less than 1 in 50,000.

### Genome annotation

Genes were identified using two gene modeling programs, Glimmer [42] and Critica [43] as part of the Oak Ridge National Laboratory genome annotation pipeline. The two sets of gene calls were combined using Critica as the preferred start call for genes with the same stop codon. Genes with less than 80 amino acids which were predicted by only one of the gene callers and had no Blast hit in the KEGG database at 1e-05, were deleted. This was followed by a round of manual curation to eliminate obvious overlaps. The predicted CDSs were translated and used to search the National Center for Biotechnology Information (NCBI) nonredundant database, UniProt, TIGRFam, Pfam, PRIAM, KEGG, COG, and InterPro databases. These data sources were combined to assert a product description for each predicted protein. Non-coding genes and miscellaneous features were predicted using tRNAscan-SE [44], TMHMM [45], and signalP [46]. Additional gene prediction analysis and manual functional annotation was performed within the Integrated Microbial Genomes (IMG) platform developed by the Joint Genome Institute, Walnut Creek, CA, USA [47].

## Genome properties

**Figure 3:**
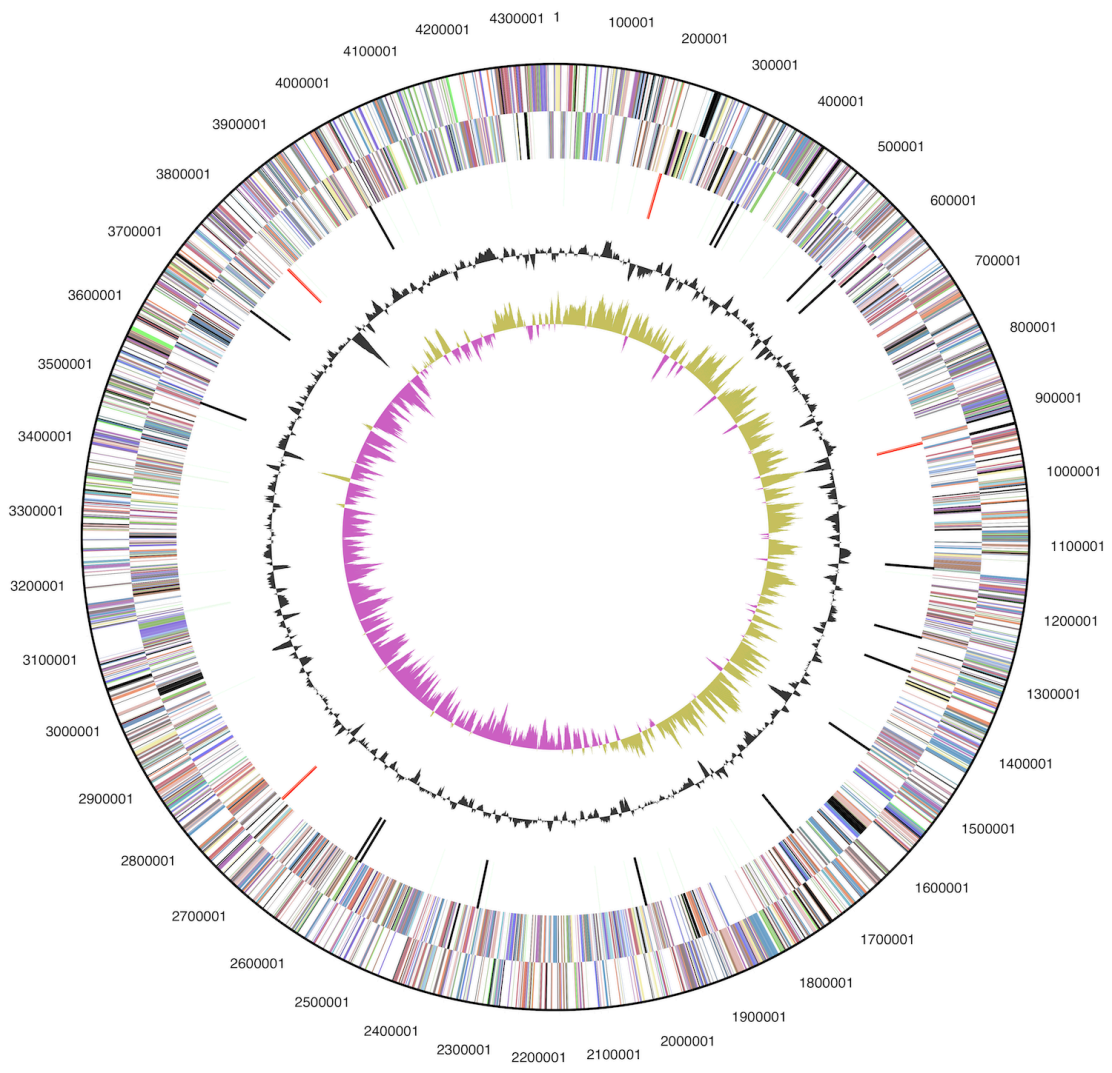
Graphical circular map of the chromosome (plasmid map not shown). From outside to the center: Genes on forward strand (color by COG categories), Genes on reverse strand (color by COG categories), RNA genes (tRNAs green, rRNAs red, other RNAs black), GC content, GC skew.

The genome consists of a 4,352,825 bp long chromosome with a 65% G+C content and a 53,732 bp plasmid with 60% G+C content (Table 3 and Figure 3). Of the 3,933 genes predicted, 3,850 were protein-coding genes, and 83 RNAs; nine pseudogenes were also identified. The majority of the protein-coding genes (72.7%) were assigned a putative function while the remaining ones were annotated as hypothetical proteins. The distribution of genes into COGs functional categories is presented in Table 4.

**Table 3:** Genome Statistics

**Attribute**	**Value**	**% of Total**
Genome size (bp)	4,406,557	100.00%
DNA coding region (bp)	3,911,312	88.76%
DNA G+C content (bp)	2,880,951	65.38%
Number of replicons	2	
Extrachromosomal elements	1	
Total genes	3,933	100.00%
RNA genes	83	2.11%
rRNA operons	4	
Protein-coding genes	3,850	97.89%
Pseudo genes	9	0.23%
Genes with function prediction	2,861	72.74%
Genes in paralog clusters	518	13.17%
Genes assigned to COGs	3,048	77.50%
Genes assigned Pfam domains	3,235	82.25%
Genes with signal peptides	776	19.73%
Genes with transmembrane helices	734	18.66%
CRISPR repeats	13	

**Table 4:** Number of genes associated with the general COG functional categories

**Code**	**value**	**%age**	**Description**
J	159	4.6	Translation, ribosomal structure and biogenesis
A	1	0.0	RNA processing and modification
K	236	6.9	Transcription
L	136	4.0	Replication, recombination and repair
B	2	0.1	Chromatin structure and dynamics
D	36	0.9	Cell cycle control, cell division, chromosome partitioning
Y	0	0.0	Nuclear structure
V	56	1.6	Defense mechanisms
T	271	7.9	Signal transduction mechanisms
M	204	5.9	Cell wall/membrane biogenesis
N	121	3.5	Cell motility
Z	0	0.0	Cytoskeleton
W	0	0.0	Extracellular structures
U	69	2.1	Intracellular trafficking and secretion, and vesicular transport
O	127	3.7	Posttranslational modification, protein turnover, chaperones
C	228	6.6	Energy production and conversion
G	173	5.0	Carbohydrate transport and metabolism
E	341	9.9	Amino acid transport and metabolism
F	69	2.0	Nucleotide transport and metabolism
H	160	4.7	Coenzyme transport and metabolism
I	126	3.7	Lipid transport and metabolism
P	222	6.5	Inorganic ion transport and metabolism
Q	67	2.0	Secondary metabolites biosynthesis, transport and catabolism
R	367	10.7	General function prediction only
S	261	7.6	Function unknown
-	885	22.5	Not in COGs
